# Management of diabetic kidney disease: where do we stand?: A narrative review

**DOI:** 10.1097/MD.0000000000033366

**Published:** 2023-03-31

**Authors:** Devada Sindhu, Gaurav Shekhar Sharma, Damodar Kumbala

**Affiliations:** a Department of Nephrology, AIIMS Rishikesh, Dehradun, India; b Diagnostic and Interventional Nephrologist, Renal Associates of Baton Rogue, Baton Rogue, LA.

**Keywords:** diabetes mellitus, diabetic kidney disease, diabetic nephropathy

## Abstract

Diabetic kidney disease is the leading cause of chronic kidney disease and end-stage renal disease. The pathogenesis and risk factors for the development of diabetic kidney disease are complex and multifaceted, resulting in glomerular hypertrophy, tubulointerstitial inflammation, and fibrosis. The clinical staging progresses over 5 stages from early hyperfiltration to overt nephropathy. Primary prevention like glycaemic control, control of blood pressure, treatment of dyslipidemia and lifestyle modifications have shown promising benefits. Despite widespread research, very few drugs are available to retard disease progression. More literature and research are needed to fill these lacunae. We carried out a literature search focusing on newer updates in diabetic kidney disease pathophysiology, diagnosis and management using a PubMed search through the National library of medicine using keywords “Diabetic kidney disease,” and “Diabetic nephropathy” till the year 2022. We have summarized the relevant information from those articles.

**Key messages**

Studies in diabetes kidney disease in the last few years have shown many new pathophysiological pathways and corresponding newer treatment options. A review of these updates would guide us in better patient management.

## 1. Introduction

The major cause of end-stage kidney disease (ESKD) worldwide is diabetes mellitus (DM).^[1]^ It can cause damage to several organ systems, including the urinary tract. The damage to the urinary system may be due to diabetic nephropathy, primary glomerular diseases, recurrent acute pyelonephritis, renal tubular acidosis, papillary necrosis, diabetic cystopathy etc.

In 2021, the International Diabetic Federation anticipated that roughly 537 million individuals (20–79 years) are living with diabetes worldwide. India was found to be the second most prevalent nation, after China. The approximate number of diabetic adults in India is 73 million.

The prevalence of microalbuminuria and macroalbuminuria among the microvascular consequences of DM is approximately 30% to 35% in both types of diabetes.^[2]^

Numerous risk factors for diabetic nephropathy can be categorized as modifiable and non-modifiable. While the former includes poor glycaemic and blood pressure control, high blood cholesterol, smoking, presence of diabetic retinopathy, use of oral contraceptives, obesity, hyperuricemia, etc, the latter consists of the male sex, positive family history for DM, the onset of Type 1 diabetes mellitus (T1 DM) before the age of 20 years, certain ethnic groups like African Americans, Mexican Americans, Pima Indians, South Asian immigrants settled in the United Kingdom etc.^[2–4]^

## 2. Diagnosis

The clinical assessment of diabetic kidney disease (DKD) is based on clinical parameters such as the duration of DM and edematous illness, the presence of diabetic retinopathy (DR), and the measurement of eGFR and albuminuria. DKD is recognized clinically by a persistently high urinary albumin-to-creatinine ratio ≥ 30 mg/g and/or unrelenting reduction in eGFR < 60 mL/min/1.73 m^2^. For albuminuria or low eGFR to be confirmed, two abnormal values must be taken at least three months apart.^[5]^

The American Diabetes Association (ADA) Standards of Medical Care in Diabetes 2022 recommend that screening for DKD should be performed at least once a year for patients with T1 DM beginning 5 years after diagnosis and in all patients with type 2 diabetes mellitus (T2 DM) at the first visit.^[6]^

Amongst patients with DM and renal involvement (in the form of proteinuria and/or failure), the following is the pattern of presentations-

- 60% – classic Kimmelstiel–Wilson disease (large kidneys, heavy proteinuria)
- 13% – an atypical presentation consistent with ischemic nephropathy (shrunken kidneys with no major proteinuria)
- 27% – had a known primary kidney disease with concomitant or superimposed diabetes.

## 3. Management

Management of DKD includes prevention of the onset of albuminuria i.e., Kidney Disease initiative: Global outcome (KDIGO) stage A1 (Primary Prevention), retarding the progression from KDIGO stage A1 to stage A2/A3 (Secondary Prevention) and treatment involved in ESKD.^[7]^

### 3.1. Primary prevention

The most important modality for Primary Prevention is strict glycemic control in patients with Type 1 DM and adequate glycemic control in those with T2 DM. Other therapy modalities for Primary Prevention include blood pressure control, treatment of dyslipidemia, and lifestyle adjustments.^[7]^

#### 3.1..1. Glycemic control.

The Diabetes Control and Complications Trial was the seminal study that demonstrated that strict glycemic control lowers the risk for microalbuminuria and impaired glomerular filtration rate (GFR) in patients with Type 1 DM. Patients receiving intensive therapy (mean HbA1c 7%) had a 35% to 45% lower risk for the development of microalbuminuria compared with the control group (mean HbA1c 9%).^[8]^

An important study in T2 DM was the United Kingdom Prospective Diabetes Study (UKPDS). The mean achieved HbA1c was 7.0% in the intense control group while it was 7.9% in the standard group. The relative risk reduction for the emergence of microalbuminuria was 24% after nine years of intensive therapy. Although the glycemic control was lost significantly earlier in the study, these patients demonstrated a reduced risk of vascular complications and a lower all-cause mortality throughout a subsequent ten-year period of follow-up.^[9]^

Since the UKPDS, three large trials, Action in Diabetes and Vascular Disease: Perindopril and Indapamide Modified Release Controlled Evaluation, Action to Control Cardiovascular Risk in Diabetes (ACCORD), and VA Diabetes Trial, have demonstrated that intensive control of blood sugars is of unproven benefit in patients with Type2 DM.

In its 2022 guidelines, the ADA suggests a target HbA1c of 7.0% to stop or slow the progression of the microvascular consequences of diabetes, including DKD in both Type 1 and Type 2 DM patients (ADA).^[6]^

#### 3.1.2. Blood pressure control.

The prevention of DKD, DR, and cardiovascular disease in diabetic individuals depends on the early management of hypertension. In individuals with hypertension who also have diabetes, the first-line antihypertensive drugs are angiotensin-converting enzyme inhibitors (ACEi) or angiotensin receptor blockers (ARBs).^[7,10]^ Renin angiotensin aldosterone blockade plays an important role in hypertension control and in the prevention of the progression of DKD. ACEi decreases the production of Ang II, which is a potent vasoconstrictor, leading to lower intraglomerular pressure and reduced glomerular hypertension. They also decrease the glomerular permeability to urinary albumin leading to decreased proteinuria. ARBs act by blocking Ang II type 1 receptor. This blockade may lead to a further increase in the synthesis of Ang II which binds to intrarenal AT_2_ receptors, resulting in decreased blood pressure and reduced renal interstitial fibrosis.^[11]^

Two key studies done in patients with T1 DM to assess the response of RAAS blockade on the advancement of both DN and DR with time, were the Renin-Angiotensin-Aldosterone System Study (RAAS) and the Diabetic Retinopathy Candesartan Trials. Both had found that with time, the progression of DR had slowed down but there was no effect on the progression of DN.^[12,13]^

The Bergamo Nephrologic Diabetes Complications Trial, which compared an ACEi versus Calcium channel blockers and found that there was less progression to the stage of microalbuminuria with the former as compared to the latter, was the landmark study that had demonstrated the beneficial effect of RAAS inhibitors in slowing the evolution of DN in patients with T2 DM.^[14]^

The Randomized Olmesartan and Diabetes Microalbuminuria Prevention trial, another significant study on the effect of ARBs on the development of nephropathy in T2 diabetic patients, demonstrated that an ARB could delay or prevent the onset of microalbuminuria in comparison to a placebo.^[15]^

In order to lower cardiovascular disease mortality and decrease the course of CKD, ADA 2022 recommends that blood pressure objectives for all normoalbuminuric diabetics be below 140/90 mm Hg. A lower blood pressure goal (130/80 mm Hg) is advised after albuminuria develops.^[6]^

#### 3.1.3. Treatment of dyslipidemia.

There are few clinical studies on the effectiveness of lipid-lowering alone in preventing DN.

The Action to Control Cardiovascular Risk in Diabetes (ACCORD)-Lipid trial and the Diabetes Atherosclerosis Intervention Study have both demonstrated a slower progression to microalbuminuria.

According to the Diabetes Atherosclerosis Intervention Study, fenofibrate significantly reduced the rate of progression from normoalbuminuria to microalbuminuria in T2 diabetes patients at 3 years compared to the placebo group.^[13]^

The Simvastatin and Fenofibrate Combination Medicine Study (ACCORD-Lipid) looked into the effects of this combination therapy. Simvastatin with fenofibrate was found to considerably slow the development of microalbuminuria in T2 DM patients as compared to the control group (simvastatin alone).^[16]^

##### 3.1.3.1 Other interventions.

Lifestyle changes such as weight reduction, exercise, and smoking cessation have been found beneficial. Dietary modification such as salt restriction (Sodium intake < 2 g of elemental sodium or < 5 g of sodium chloride per day) has also been recommended.^[17]^

### 3.2. Secondary prevention

#### 3.2.1. Hypertension control.

It is involved in retarding the progression from KDIGO stage A1 to A2/A3. Blood pressure control is indeed the most important strategy in the treatment of DKD and prevention of the onset of KDIGO stage A2.^[1]^

##### 1.3.2.1. Blood pressure control.

Patients with urine albumin excretion of more than 30 mg per 24 hours should have a target blood pressure of 130/80 mm Hg or less, and all diabetic patients should have a target blood pressure of 140/90 mm Hg or less.^[6]^ Additionally, it advises patients with DM and hypertension who have urinary albumin-to-creatinine ratios below 300 mg/g creatinine and/or eGFRs below 60 mL/min/1.73 m^2^ to use ACEi or ARBs as first-line medications.

Two landmark studies were done in patients with T2 DM, which showed the benefit of intensive blood pressure control over less stringent control were the UKPDS and the Appropriate Blood Pressure Control in Diabetes trial.^[18]^ Both proved that there was a reduction in the development of microalbuminuria in those patients whose blood pressure was controlled more strictly.^[19]^ However, the Action to Control Cardiovascular Risk in Diabetes (ACCORD) blood pressure trial (ACCORD BP) had depicted that there was no reduction in the rate of major cardiovascular events in those Type2 diabetic patients whose blood pressure was controlled more intensively (Systolic blood pressure < 120 mm Hg) as compared to those whose blood pressure BP was kept on a higher side (Systolic blood pressure < 140 mm Hg).^[16]^

It is important to note that the RAAS blockade in patients with DKD, by either ACEi or with ARBs provides renoprotection that is independent of blood pressure reduction.^[8]^

Virtually all studies done in Type1 diabetic patients with nephropathy were based on ACEi, not ARBs. In a meta-analysis of 12 placebo-controlled trials enrolling normotensive patients with Type1 DM and microalbuminuria treated with ACEi, there was a 60% decline in evolution to macroalbuminuria and there was also a threefold rise in regression to normoalbuminuria.^[2]^

The serum creatinine concentration has been seen to increase by up to 30% in proteinuric patients with renal impairment after taking RAAS blockers. If the increase in serum creatinine concentration occurs within 4 weeks of starting ACEi or ARBs and is greater than 30%, they should be stopped and this should raise the possibility of underlying renal artery stenosis. Measures to lower serum potassium levels should be used to treat hyperkalemia brought on by ACEi or ARBs rather than lowering the dose or terminating ACEi or ARBs right away.^[1]^

As additional antihypertensive medications, dihydropyridine calcium channel blockers such as nisoldipine, nifedipine, amlodipine, etc., may be utilized; however, these medications have not been demonstrated to reduce albuminuria or to slow the progression of the renal diseases.^[17]^

#### 3.2.2. Other anti-proteinuric modalities.

A low-sodium diet (<2.0 g sodium/d) may help. For lowering blood pressure and reducing proteinuria, a combination of a loop diuretic or thiazide diuretic plus RAAS blockers may be superior to either kind of treatment alone. SARAs, or selective aldosterone receptor antagonists, have also been proven to reduce proteinuria when administered alone and to increase it when combined with ACEi or ARBs. The FIDELIO-DKD trial demonstrated that finerenone therapy resulted in decreased risks of cardiovascular events and CKD progression than placebo.^[20]^ Some investigations have demonstrated that non-dihydropyridine calcium channel blockers, such as diltiazem and verapamil, have positive antiproteinuric effects.^[4]^

#### 3.2.3. Glycemic control.

In T2 DM patients with DKD (A1/A2), the KDIGO 2020 suggests an individual HbA1c target range from 6.5% to 8%. An HbA1c target of 7% is advised by ADA 2022. Additionally, it indicates that lowering the HbA1c level would be advantageous if it could be done so without substantial hypoglycemia or other negative treatment side effects.^[6]^

For T1 DM, ADA 2022, recommends multiple daily injections of prandial and basal insulin or continuous subcutaneous insulin infusion as first-line therapy. The use of sodium-glucose cotransporter 2 inhibitors (SGLT-2i), which prevent sodium and glucose uptake in the proximal tubule and cause natriuresis and glucosuria, to treat Type 1 diabetics is still relatively new. However, the National Institute for Health and Care Excellence advised the use of sotagliflozin in 2020 for T1 DM patients when insulin alone is insufficient to control glycemia.^[21]^ Another agent, Pramlintide can be used if renal functions are normal but limited experience is available regarding its use in CKD and is therefore not recommended in this subset of patients.

For Type 2 diabetics with DKD, large studies (e.g., Kumamoto study, Action in Diabetes and Vascular Disease: Perindopril and Indapamide Modified Release Controlled Evaluation, ACCORD) advocate that intensive glycemic control may provide some renoprotection but does not safeguard against macrovascular complications. Thus, anti-diabetic therapy must be tailored to T2 DM.^[4]^ SGLT2i are relatively new drugs that have been permitted for the treatment of T2 DM. Sodium-glucose cotransporter 2 proteins are expressed in the proximal convoluted tubules of the kidney. They are responsible for approximately 90% of the filtered glucose reabsorption. Inhibition of this causes glucosuria leading to better glycaemic control. Together with glucosuria, SGLT2i also cause natriuresis leading to a better BP control. Natriuresis increases sodium delivery to the juxta-glomerular apparatus in the distal nephron, which through tubule-glomerular feedback causes afferent arteriolar constriction, thereby reducing glomerular hyperfiltration seen in diabetic nephropathy.^[22]^ Recently, two trials designed as cardiovascular safety studies- Empagliflozin, Cardiovascular Outcomes, and Mortality in T2 DM and Canagliflozin Cardiovascular Assessment Study, have revealed noteworthy cardiovascular risk reduction when these are used for the management of T2 DM. In these cardiovascular outcome trials, these agents were found to have positive effects on kidney outcomes, namely albuminuria reduction and a reduction in the occurrence of a composite renal outcome.^[9]^ In patients with T2 DM with CKD, the CREDENCE (Canagliflozin and Renal Events in Diabetes with Established Nephropathy Clinical Evaluation) trial showed that long-term administration of canagliflozin conferred better renal outcomes which include slower progression to ESKD.^[23]^ Similarly in the Dapagliflozin and Prevention of Adverse Outcomes in Chronic Kidney Disease trial, the risk of sustained decline in the estimated GFR (eGFR) of at least 50%, ESKD or death from renal or cardiovascular causes was found to be significantly lower with dapagliflozin than with placebo.^[24]^

Based on these studies, for T2 DM, KDIGO 2020 and ADA 2022 recommends Metformin and SGLT2i (or gliflozins) as first-line treatment modalities. The patients should be counseled regarding the potential adverse reactions or side effects e.g., diabetic ketoacidosis, urinary tract infection, volume contraction, acute kidney injury, bone fractures etc of SGLT2i.^[24]^ It is important to note that these agents are contraindicated in patients with an eGFR < 30 mL/min/1.73m^2^ (1). Long-acting GLP-1 receptor agonists (Exenatide, Liraglutide, Lixisenatide, Semaglutide, etc) are used as the preferred second-line agents in patients with Type2 DM and CKD.

#### 3.2.4. Treatment of dyslipidemia.

In type 2 diabetics patients with non–dialysis-dependent DN, treatment with statins (dose based on creatinine clearance) provides substantial cardiovascular benefit. In the UKPDS, elevated triglycerides were independently associated with albuminuria in T2 diabetic patients Field.^[25]^ Thus, interventions aimed at improving all lipid targets are required. The largest trial performed on the role of aggressive lipid reduction in diabetes was the ACCORD Lipid trial, in which intensive lipid lowering with simvastatin and fenofibrate versus simvastatin and placebo was carried out out.^[26]^ The addition of fenofibrate did not affect the primary CV outcome of the trial, nor was there a difference in the incidence of ESKD or dialysis requirement. There was, however, a reduction in both microalbuminuria and overt proteinuria in the intensive group. Thus, intensive lipid lowering with simvastatin and fenofibrate may reduce proteinuria but may not prevent death or dialysis in T2 DM.^[4]^

#### 3.2.5. Dietary protein restriction.

Dietary protein restriction may alleviate uremic symptoms in patients at or approaching ESKD. However, it is of uncertain benefit in the treatment of DN. Small trials have shown a low-protein diet (0.8 g/kg/d) to reduce proteinuria significantly with increased plasma albumin in Type 2 diabetic patients with moderately increased albuminuria.

Other complications of CKD per se are to be managed according to standard guidelines.

### 3.3. Treatment in end-stage kidney disease.

Patients who are being treated with hemodialysis and peritoneal dialysis should consume a protein intake of 1.2 and 1.3 g/kg/d respectively, with a calorie intake of 30 to 35 Kcal/kg/d, apart from the diabetic diet.^[1]^

A diabetic patient with ESKD has multiple options for renal replacement therapy (RRT), which include Hemodialysis, Peritoneal dialysis, and Transplantation. Out of these, transplantation is the best modality. For a patient with T1 DM and ESKD, transplantation can be of three types – Kidney only, Simultaneous Pancreas with Kidney (SPKT), and Pancreas after Kidney (PAKT). Out of all these, the best results are with SPKT.^[7,27]^ Timely creation of vascular access in these patients is crucial. It is recommended that it should be initiated when eGFR is 20 to 25 mL/min/1.73 m^2^.^[7]^

In the past, RRT was initiated early in diabetic patients to decrease symptoms of uremia and diabetes. It is yet unknown when maintenance dialysis should start for people with ESKD. The Initiating Dialysis Early and Late research, a randomized trial that examined the impact of dialysis beginning at two distinct levels of kidney function, found no appreciable differences between the two treatment groups in terms of survival or other patient-centered outcomes. Additionally, a systematic evaluation of the literature found no differences in mortality between patients with diabetes and those without diabetes between early (higher eGFR) and late (lower eGFR) starts of RRT. Thus, regardless of the existence or lack of, RRT should begin based on the same principles in all patients.^[28,29]^

### 3.4. Emerging treatment modalities

**Figure 1.**
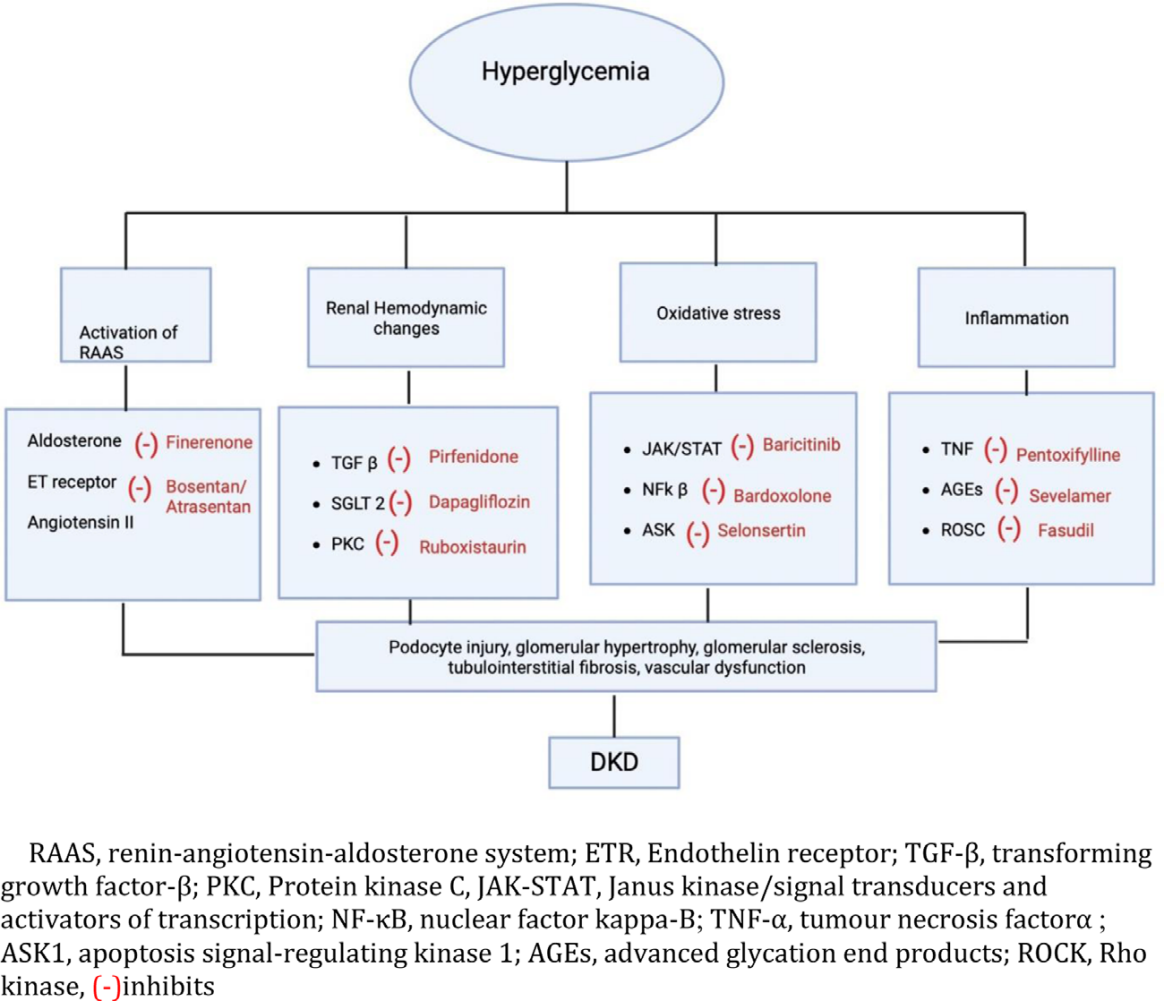
Overview of pathophysiology and mechanism of action of newer drugs in the management of diabetic kidney disease. (-) = inhibits, AGEs = advanced glycation end products, ASK1 = apoptosis signal-regulating kinase 1, ETR = endothelin receptor, JAK-STAT = Janus kinase/signal transducers and activators of transcription, NF-κB = nuclear factor kappa-B, PKC = protein kinase C, RAAS = renin-angiotensin-aldosterone system, ROCK = rho kinase, TGF-β = transforming growth factor-β, TNF-α = tumour necrosis factor α.

Numerous treatments targeting several proposed molecular mechanisms of cell injury in patients with DKD have been tried. These can be arbitrarily classified into drugs that affect vasculature, anti-inflammatory agents, and drugs targeting oxidative stress.^[30]^ See (Fig. 1), which depicts the pathophysiology and mechanism of action of various drugs. Amongst the drugs that affect the vasculature, endothelin receptor antagonists (Atrasentan, Bosentan) have been shown to reduce albuminuria in patients with DKD. The Tie-2 activators (AKB-9778), which are inhibitors of vascular endothelial-protein tyrosine phosphatase (VE-PTP) have shown renoprotective effects in DKD patients.^[30,31]^

Amongst the anti-inflammatory agents, an inhibitor of phosphokinase C-Ruboxistaurin (in phase III RCT) has been found to improve glomerular filtration and decrease proteinuria. Nuclear factor erythroid-derived 2-related factor 2 (Nrf2) activator (Bardoxolone methyl) is another anti-inflammatory agent which protects the renal tubular epithelial cells in DKD by improving redox balance and mitochondrial function and inhibiting inflammation.^[30]^

JAK-STAT pathway inhibitors (Baricitinib) have been found to have a noticeable effect on decreasing proteinuria, mesangial dilation, decreased podocyte density, and glomerulosclerosis. The phosphodiesterase inhibitors (Pentoxifylline and Cilostazol) have been found to have anti-inflammatory and immunomodulatory effects in DKD and were found to reduce the decline of eGFR and significantly decrease proteinuria in T2DM patients with CKD stages 3 to 4.^[31]^

Amongst the drugs targeting oxidative stress, NOX 1/4 inhibitors and Xanthine oxidase inhibitors (allopurinol) were found to have a renoprotective effect. Inhibitors of advanced glycation end products have been studied in DKD stages 2 to 4. Sevelamer, a commercially used non-calcium phosphorus binder, decreased the expression of advanced glycation end products and thereby conferred a renal protective effect in DKD patients.^[32]^ See (Table 1), which depicts the summary of all clinical trials in newer drugs and their important outcomes. Many new trials are underway and some of these agents will probably be approved for clinical use within the next few years.^[30,33,34]^

**Table 1:** Newer drugs in the management of diabetic kidney disease.

Drug group	Drug name	Study	Main renal outcome
SGLT2 inhibitors	Canaglifozin	CREDENCE	Comparing canaglifozin to a placebo, it reduced UACR by 31%.
		CANVAS^[35]^	When compared to the placebo group, the canagliflozin group experienced less frequent the composite endpoint of persistent doubling of serum creatinine, ESRD, and death from renal causes.
	Empagliflozin	EMPA-REG OUTCOME^[36]^	When compared to placebo, empaglifozin reduced the relative risk of developing albuminuria, incident or worsening nephropathy.
		EMPA-CKD^[37]^	13.1% of those using empagliflozin experienced kidney disease progression.
	Dapagliflozin	DECLARE-TIMI 58^[38]^	In the entire cohort, dapagliflozin reduced the renal-specific outcome compared to placebo.
		DAPA-CKD^[39]^	Dapagliflozin and placebo had equivalent mean rates of eGFR reduction while dapagliflozin decreased the urine albumin-to-creatinine ratio by 26% more.
Enhancing GLP1 expression	Liraglutide	LEADER^[40]^	Liraglutide slowed the development and progression of DKD than placebo.
	Lixisenatide	ELIXA^[41]^	Lixisenatide reduced albuminuria and had decreased UACR compared to placebo.
	Dulaglutide	REWIND^[42]^	Dulaglutide had reduced albuminuria and the decline of eGFR.
Inhibiting PKC	Ruboxistaurin	RCT (phase 2/3)^[43]^	Ruboxistaurin had lower rates of progression and development of albuminuria compared to placebo.
Inhibiting RAAS	Finerenone	FIDELIO-DKD^[20]^	Compared to a placebo, finerenone had lower albuminuria in DKD patients.
Inhibiting JAK-STAT pathway	Baricitinib	RCT (phase 2)^[44]^	Baricitinib Reduced albuminuria compared to proteinuria.
Inhibiting PDE	Pentoxifylline	PENFOSIDINE STUDY^[45]^	Pentoxifylline had lesser decline in eGFR and decreased albuminuria compared to placebo.
	Cilostazol	RCT (Phase 4)^[46]^	Cilostazole decreased albuminuria and cytokines compared to placebo.
ETR antagonism	Atrasentan^[46]^	RCT (phase 3) SONAR^[47]^	Atrasentan had lower albuminuria compared to placebo.
Inhibiting TGF-β	Pirfenidone^[48]^	RCT (phase 3)	Pirfenidone decreased albuminuria compared to placebo.
VDR Activator	Paricalcitol^[49]^	RCT (phase 4)	Paricalcitol had lower albuminuria than placebo.

AGEs = advanced glycation end products, CANVAS = CANagliflozin Cardiovascular Assessment Study (CANVAS), CREDENCE = Canagliflozin and Renal Events in Diabetes with Established Nephropathy Clinical Evaluation, DAPA-CKD = Dapagliflozin and Prevention of Adverse outcomes in Chronic Kidney Disease, DECLARE-TIMI 58 = The Dapagliflozin Effect on Cardiovascular Events–Thrombolysis in Myocardial Infarction 58, ELIXA = The Evaluation of Lixisenatide in Acute Coronary Syndrome, EMPA-CKD = Study of Heart and Kidney Protection with Empagliflozin, EMPA-REG OUTCOME = Empagliflozin Cardiovascular Outcome Event Trial in Type 2 Diabetes Mellitus Patients–Removing Excess Glucose (EMPA-REG) OUTCOME, ETR = endothelin receptor, FIDELIO-DKD = Efficacy and Safety of Finerenone in Subjects With Type 2 Diabetes Mellitus and Diabetic Kidney Disease, JAK-STAT = Janus kinase/signal transducers and activators of transcription, LEADER = Liraglutide Effect and Action in Diabetes: Evaluation of Cardiovascular Outcome Results, PDE = phosphodiesterase, PKC = protein kinase C, RAAS = renin-angiotensin-aldosterone system, REWIND = The Researching cardiovascular Events with a Weekly INcretin in Diabetes, TGF-β = transforming growth factor-β, UACR = urine albumin-creatinine ratio, VDR = vitamin D receptor.

## 4. Conclusion

The overall prevalence of microalbuminuria and macroalbuminuria is around 30% to 35% in both types of diabetes. DKD is recognized clinically by a persistently high urinary albumin-to-creatinine ratio ≥ 30 mg/g and/or unrelenting reduction in eGFR < 60 mL/min/1.73 m^2^. Primary Prevention includes glycaemic control, control of blood pressure, treatment of dyslipidemia and lifestyle modifications. ADA 2022 recommends a target blood pressure of 140/90 mm Hg or less for all diabetic patients, and 130/80 mm Hg or less for patients with urine albumin excretion of greater than 30 mg per 24 hours. RAAS blockade in patients with DKD, by either ACEi or with ARBs provides renoprotection that is independent of blood pressure reduction. KDIGO 2020 and ADA 2022 recommend Metformin and an SGLT2i (or gliflozins) as first-line treatment modalities of DM in DKD.

## Author contributions

**Conceptualization:** Devada Sindhu, Gaurav Shekhar Sharma, Damodar Kumbala.

**Data curation:** Devada Sindhu, Gaurav Shekhar Sharma, Damodar Kumbala.

**Formal analysis:** Devada Sindhu, Gaurav Shekhar Sharma, Damodar Kumbala.

**Investigation:** Devada Sindhu, Gaurav Shekhar Sharma, Damodar Kumbala.

**Methodology:** Devada Sindhu, Gaurav Shekhar Sharma, Damodar Kumbala.

**Project administration:** Devada Sindhu, Gaurav Shekhar Sharma, Damodar Kumbala.

**Resources:** Devada Sindhu, Gaurav Shekhar Sharma, Damodar Kumbala.

**Software:** Devada Sindhu, Gaurav Shekhar Sharma.

**Supervision:** Devada Sindhu, Gaurav Shekhar Sharma, Damodar Kumbala.

**Validation:** Devada Sindhu, Gaurav Shekhar Sharma, Damodar Kumbala.

**Visualization:** Devada Sindhu, Gaurav Shekhar Sharma, Damodar Kumbala.

**Writing – original draft:** Devada Sindhu, Gaurav Shekhar Sharma, Damodar Kumbala.

**Writing – review & editing:** Devada Sindhu, Gaurav Shekhar Sharma, Damodar Kumbala.
